# Spontaneous splenic rupture: A rare complication of concurrent malaria and dengue infections – A case report

**DOI:** 10.1016/j.ijscr.2024.110783

**Published:** 2024-12-27

**Authors:** Hassan Elmi Moumin, Abdirahman Omer Ali, Ahmed Abdi Aw Egge, Mohamoud Hashi Abdi, Abdillahi Elmi Moumin, Abdisalam Hassan Muse

**Affiliations:** a College of Health Sciences, School of Medicine and Surgery, Amoud University, Borama, Somalia; b School of Postgraduate Studies and Research, Amoud University, Amoud Valley, Borama 25263, Somalia; c Borama Regional Hospital, Surgery Department, Somalia; d Borama Regional Hospital, Radiology Department, Somalia

**Keywords:** Spontaneous splenic rupture, Co-infection, Splenectomy, Diagnostic challenges, Resource-limited settings

## Abstract

**Introduction:**

Spontaneous splenic rupture (SSR) is a rare, life-threatening complication, sometimes associated with infections like malaria and dengue fever. This case report details a unique presentation of SSR.

**Case presentation:**

A 28-year-old male in Somalia presented with fever, epigastric pain, nausea, vomiting, and body aches, consistent with malaria and dengue. Following self-discharge after initial malaria treatment, his condition deteriorated rapidly, leading to severe abdominal pain and hypotension. Laboratory tests confirmed malaria and dengue, with low hemoglobin. A CT scan revealed a large hemoperitoneum and splenic rupture requiring emergency laparotomy and splenectomy. Post-operative recovery was uneventful.

**Discussion:**

This case highlights the diagnostic challenges posed by overlapping symptoms of malaria and dengue, potentially masking SSR. The concurrent infections likely contributed to splenomegaly, increasing rupture risk. While SSR has been linked to malaria or dengue individually, this case suggests a potential synergistic effect of co-infection. The patient's self-discharge emphasizes the importance of patient education and treatment adherence. Successful surgical intervention underscores the critical role of prompt medical care.

**Conclusion:**

This is the first reported case of SSR secondary to concurrent *Plasmodium falciparum* malaria and dengue fever in Somalia. This highlights the need for improved diagnostic tools, healthcare infrastructure, and targeted public health interventions in endemic regions. Further research is crucial to understand the synergistic effect of these co-infections in inducing SSR.

## Highlights

• **Rarity:** The case represents a rare event, particularly the combination of both infections leading to SSR, and the first such reported case in Somalia.
• **Diagnostic Challenge:** The initial presentation mimicked the symptoms of both malaria and dengue, delaying diagnosis and highlighting the difficulty in differentiating SSR from other acute abdominal conditions.
• **Synergistic Effect:** The study suggests a potential synergistic effect of the co-infection in increasing the risk of splenic rupture, emphasizing the importance of considering this complication in such cases.
• **Successful Management:** Despite the initial delay, prompt surgical intervention led to a successful outcome, highlighting the importance of timely diagnosis and appropriate treatment.
• **Policy Implications:** The case underscores the need for improved diagnostic capabilities, enhanced healthcare infrastructure, effective public health interventions, patient education, and further research into the interplay between these infections and the risk of SSR.

## 1 Introduction

Spontaneous splenic rupture (SSR) is a rare, life-threatening condition that typically manifests as left upper quadrant abdominal pain and hemodynamic instability [1,2]. It is often associated with underlying pathological conditions, including infectious diseases, which can lead to acute abdominal pain and intra-abdominal hemorrhage. In particular, infections such as malaria and dengue fever have been identified as potential precipitating factors for SSR. The condition has an incidence rate of less than 0.5 %, and while it is predominantly linked to hematological disorders and inflammatory conditions, the risk increases significantly in patients with concurrent infections. In regions endemic to malaria, SSR may be precipitated by malaria infections, which affect millions globally and can lead to severe complications [2,3]. This case report presents a unique instance of SSR attributed to concurrent Plasmodium falciparum malaria and dengue fever in Somalia.

We introduce the case of a 28-year-old male patient who experienced splenic rupture attributable to Plasmodium falciparum (P. falciparum) infection (malaria) and dengue fever. This case report marks the first documented instance of spontaneous splenic rupture in Somalia. This case is presented in accordance with the SCARE 2023 guidelines [4].

## 2 Case presentation

A 28-year-old male patient presented to the outpatient clinic with a two-day history of fever, epigastric pain, nausea, vomiting, body aches, and sweating. Physical examination, despite high grade fever, was unremarkable. Basic laboratory investigations were within the normal range except malaria and dengue fever testing returned positive and oral anti-malarial medication was started. After the first dose of anti-malarial drugs, he experienced severe vomiting, leading to a refusal to continue treatment and self-discharge. Epigastric pain became more severe, vomiting persisted, and he developed chills/rigors and high-grade fever as well. He came back to the health facility, referral hospital Borama regional hospital, after 3 days. His symptoms worsened, characterized by: Day 1: Low-grade fever, mild epigastric pain, nausea, vomiting. Day 2: Persistent fever (up to 39 °C), severe epigastric pain, increased vomiting, and chills. Day 3: Severe abdominal pain, loss of appetite, and a fever of 40 °C. Day 4: he presented with diffuse abdominal pain.

## 3 Clinical findings

**Fig. 1:**
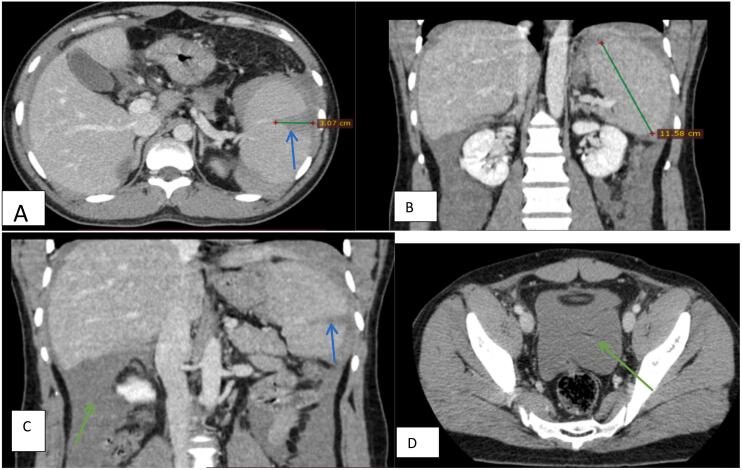
Contrast-enhanced CT of the abdomen. a) Axial, and coronal CT scan at portal venous phase shows: linear hypo dense extending from the inferior splenic capsule to the splenic cortex measuring 3 cm, (blue arrows in a and c images) *peri* splenic and splenic capsule hematoma is identified (a and c). b) Mild splenomegaly measuring 11.5 cm in CC measurement. c) hemoperitoneum around the liver and pelvic seen (orange arrows in C and D images). (For interpretation of the references to color in this figure legend, the reader is referred to the web version of this article.)

Upon examination, the patient displayed significant pallor and tachycardia, hypotension, abdominal distension with signs of fluid accumulation and peritoneal irritation. He was re-admitted to referral hospital. Upon admission, the following laboratory results were documented: Hemoglobin (Hb): 7.7 g/dL White Blood Cells (WBC): 10,000/mm^3^, Platelet Count: 151,000/mm^3^, Coagulation Profile: Normal (PT, aPTT, INR within normal limits), Creatinine: 0.9 mg/dL (normal range) Electrolytes: Sodium: 135 mEq/L (normal), Potassium: 4.0 mEq/L (normal), Chloride: 100 mEq/L (normal), Bicarbonate: 24 mEq/L (normal). Dengue fever and Malaria tests became positive. Contrast-enhanced CT of the abdomen was performed, revealing; A linear hypodense area extending from the inferior splenic capsule to the splenic cortex, measuring 3 cm. Presence of a peri-splenic and splenic capsule hematoma. Mild splenomegaly, measuring 11.5 cm in the craniocaudal dimension. Massive hemoperitoneum surrounding the liver and pelvis as shown in Fig. 1.

## 4 Diagnostic assessment

The patient was diagnosed with spontaneous splenic rupture (SSR) secondary to severe malaria and concurrent dengue fever.

## 5 Therapeutic intervention

The patient was placed on NPO (nothing by mouth) status and received; Intravenous fluids, I.V Paracetamol, metoclopramide, 2 units of Blood transfusions and Intravenous anti-malarial were given(artesunate) and Surgical side was consulted for evaluation.

**Fig. 2:**
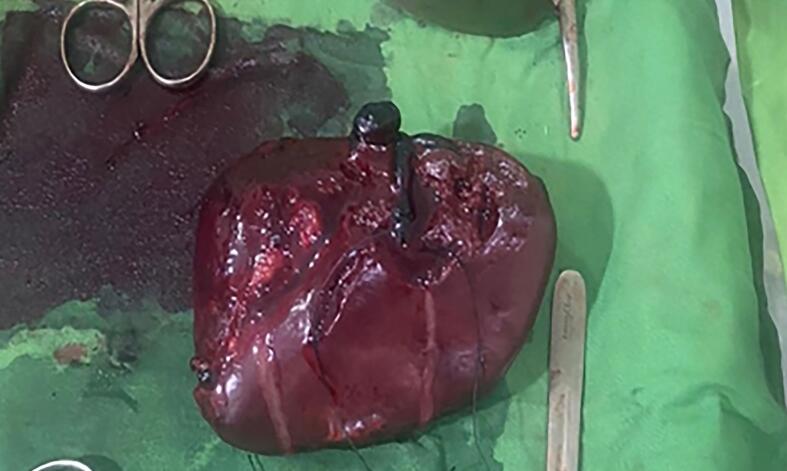
Post-splenectomy— with multiple splenic laceration.

The patient was taken to operating room and emergency laparotomy was performed. Approximately 4 l of blood were found in the general peritoneal cavity, and the spleen exhibited a rupture on the medial surface with multiple lacerations see in Fig. 2. Other intra-abdominal organs appeared normal, and a splenectomy was performed. Intraoperatively, other 3 units of whole blood were transfused.

## 6 Follow-up and outcome

On postoperative day 1, the patient was hemodynamically stable, with a hemoglobin level of 11.5 g/dL, packed cell volume (PCV) of 35 %, and WBC count of 11,000/mm^3^. He demonstrated significant clinical improvement, was able to ambulate, and had minimal drainage output. Drainage tube was removed on post-op day 5 and the patient was discharged home postoperative day 7 in stable condition. During recovery, the patient was educated about the importance of vaccinations against encapsulated organisms due to the loss of splenic function, including pneumococcal, meningococcal, and *Haemophilus influenzae* type b vaccinations.

**Fig. 3:**
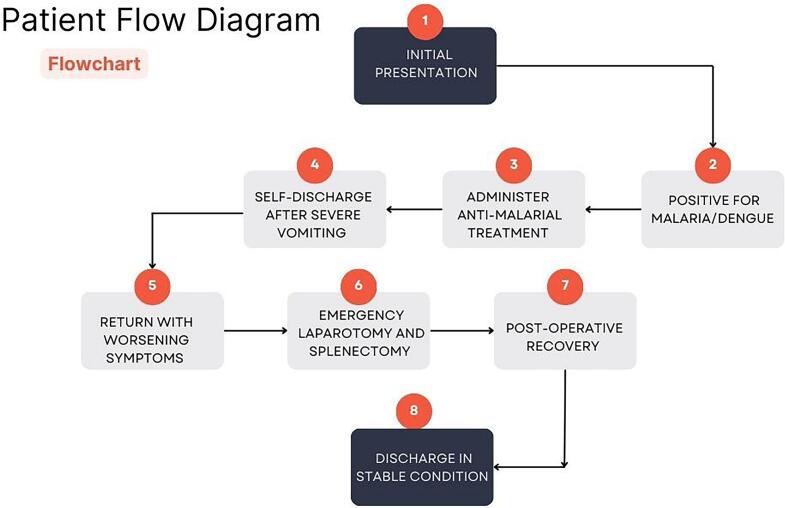
Patient flow diagram.

The patient's long-term outcome was favorable, with no immediate complications noted following discharge. However, he was advised to monitor for potential long-term complications related to splenectomy, such as increased susceptibility to infections and the need for ongoing vaccinations. Regular follow-up appointments were scheduled to monitor his recovery and overall health see in Fig. 3.

## 7 Timeline of events

- Day 0: Patient presents with fever, epigastric pain, nausea, vomiting, and body aches.
- Day 1: Initial tests confirm malaria and dengue. Start of anti-malarial treatment.
- Day 3: Patient self-discharges after experiencing severe vomiting.
- Day 6: Returns with worsening symptoms, including severe abdominal pain and hypotension.
- Day 7: Emergency laparotomy performed; splenic rupture confirmed.
- Post-operative Day 1: Patient stable; hemoglobin level improves.
- Post-operative Day 5: Drainage tube removed.
- Post-operative Day 7: Patient discharged in stable condition.

## 8 Discussion

Malaria and dengue fever share overlapping pathophysiological features that can exacerbate the severity of each other. Malaria, caused by the Plasmodium parasite, elicits an intense immune response characterized by the activation of both innate and adaptive immune systems, which can lead to splenomegaly due to increased sequestration of infected red blood cells and immune cells within the spleen [12,13]. Concurrently, dengue fever is associated with immune dysregulation and increased vascular permeability, often resulting in plasma leakage, further contributing to splenomegaly. The combination of these mechanisms may heighten the risk for SSR [[12], [13], [14]].

The splenic enlargement associated with both infections can render the organ more susceptible to rupture, particularly during episodes of increased intra-abdominal pressure or trauma. Moreover, the inflammatory cytokines released during both infections may compromise the integrity of the splenic capsule, predisposing it to rupture under stress. SSR can lead to acute abdominal pain and intra-abdominal hemorrhage, constituting a rare abdominal emergency necessitating prompt diagnosis and timely intervention to secure patient survival [[6], [7], [8],[12], [13], [14]].

The most prevalent etiological factors associated with SSR include hematological disorders (30.3 %), inflammatory conditions (20 %), infectious diseases (27.3 %), medication side effects (9.2 %), mechanical disorders (6.8 %), and indeterminate causes (6.4 %). While the mortality rate is relatively low in the absence of an underlying etiology, it may rise to as high as 12.2 % when such conditions are present [12,13]. In malaria-endemic regions, SSR may be precipitated by malaria infection itself, which is transmitted to humans through the bite of the Anopheles mosquito [10].

Malaria alone significantly impacts global health, affecting over 500 million individuals and resulting in more than 2.5 million deaths annually [11]. In 2018, the World Health Organization (WHO) documented 228 million cases of malaria worldwide, leading to 405,000 deaths, with the African region accounting for 93 % of the cases and 94 % of malaria-related fatalities. Co-infection with dengue and malaria is especially prevalent in endemic areas, where both diseases' vectors coexist [12] [13].

This case report details a rare instance of spontaneous splenic rupture (SSR) in a 28-year-old male patient, precipitated by concurrent *Plasmodium falciparum* malaria and dengue fever. The interplay between these two infections complicates the clinical picture, as both diseases can cause splenomegaly and impact platelet count, increasing the risk of hemorrhagic complications such as SSR. This represents the first documented case of SSR attributed to these dual infections in Somalia. The patient presented with a constellation of symptoms typical of both infections, initially exhibiting fever, epigastric pain, nausea, vomiting, and body aches. His condition deteriorated rapidly, culminating in severe abdominal pain, hypotension, and signs of peritoneal irritation. Diagnostic imaging confirmed a significant hemoperitoneum and splenic rupture requiring emergent splenectomy. Post-operative recovery was uneventful, highlighting the efficacy of prompt surgical intervention in managing this life-threatening complication.

This case aligns with existing literature demonstrating malaria and dengue fever as potential etiologies of SSR [[1], [2], [3], [4], [5]]. The splenomegaly resulting from both diseases likely predisposed the patient to rupture [18]. While isolated cases of SSR secondary to either malaria or dengue have been reported, this case highlights the potential synergistic effect of co-infection in increasing the risk of this severe complication.

This case contrasts with studies emphasizing the rarity of SSR even in regions with high malaria prevalence [1,16]. The concurrence of both malaria and dengue fever may represent an unusual confluence of factors leading to splenic rupture. Furthermore, while previous literature has documented cases of SSR misdiagnosed as other conditions, our patient's presentation, although initially ambiguous, ultimately led to the correct diagnosis and timely intervention [19]. The initial delay in treatment due to the patient's self-discharge after receiving the first dose of antimalarials emphasizes the critical role of patient education and adherence to medical advice in managing infectious diseases.

The treatment of co-infections like malaria and dengue presents multifaceted challenges. Clinicians must navigate the complexities of diagnosing and managing overlapping symptoms, which can lead to misdiagnosis or delayed treatment. In resource-limited settings, access to diagnostic tools may be restricted, complicating timely intervention [10,11,21,22].

Malaria treatment generally involves antimalarial medications, while dengue management focuses on supportive care to manage symptoms and prevent complications. The simultaneous administration of treatments requires careful monitoring, as certain antimalarial drugs may adversely affect patients with dengue, particularly regarding bleeding risks due to thrombocytopenia [12,13,21,22].

This case report documents a rare complication of dual infection, highlighting the need for heightened clinical awareness in endemic areas. It underscores the importance of considering SSR in the differential diagnosis of patients presenting with severe abdominal pain, particularly in regions with high prevalence of malaria and dengue. Additionally, this case contributes to the limited data on SSR secondary to co-infection, enriching our understanding of the complex interplay between infectious diseases and the risk of splenic rupture.

This case study has implications for several Sustainable Development Goals (SDGs). The successful management of this patient contributes to SDG 3 (Good Health and Well-being) by addressing a potentially fatal complication of infectious diseases prevalent in low-income countries. The case also indirectly relates to SDG 1 (No Poverty) and SDG 10 (Reduced Inequalities), as access to timely diagnosis and surgical care remains a significant challenge in resource-limited settings. Furthermore, increased awareness and improved health infrastructure can contribute to reducing the morbidity and mortality associated with malaria and dengue, aligning with SDG 3's target of ending preventable deaths by 2030. Further research into the specific mechanisms underlying the synergistic effect of co-infection in inducing SSR is warranted to improve preventative strategies and treatment guidelines.

## 9 Conclusion

This case report presents a rare instance of spontaneous splenic rupture (SSR) in a patient with concurrent *Plasmodium falciparum* malaria and dengue fever, the first such documented case in Somalia. The patient's rapid clinical deterioration, initially masked by symptoms common to both infections, highlights the diagnostic challenge posed by such co-infections. Successful management, including prompt surgical intervention, resulted in a favorable outcome. The case underscores the importance of considering SSR in the differential diagnosis of patients presenting with severe abdominal pain in malaria- and dengue-endemic regions, particularly when co-infection is suspected. The synergistic effect of these dual infections in increasing the risk of SSR warrants further investigation. While the rarity of this complication emphasizes the need for heightened clinical awareness, the successful outcome demonstrates the importance of timely diagnosis and appropriate surgical management.

## Consent for publication statement

Written informed consent was obtained from the patient for publication and any accompanying images. A copy of the written consent is available for review by the Editor-in-Chief of this journal on request.

## Patient consent

Written informed consent was obtained from the patient for publication of this case report and accompanying images. A copy of the written consent is available for review by the Editor-in-Chief of this journal on request.

## CRediT authorship contribution statement

Dr. abdirahman Omer ali Dr. Ahmed Abdi Aw Egge and Abdillahi Elmi Moumin individuals contributed to taking history and providing care to the patient throughout her hospital stay. Hassan Elmi Moumin was done splenectomy. Additionally, Dr. abdirahman Omer ali, Abdisalam Hassan Muse, abdirahman omer ali and Dr. Ahmed Abdi Aw Egge contributed to the development of the manuscript. Dr. Mohamoud Hashi Abdi: Radiologist.

## Guarantor

Dr. Abdirahman Omer Ali.

## Research Registration Number

Not applicable.

## Ethical considerations

The study protocol, case investigation, and consent form were thoroughly examined by the institutional review board of the College of Health Sciences at Amoud University. They granted approval for the study, along with the Ministry of Health and Borama Regional Hospital in Awdal Region, Somaliland (BRHH-160/2024). Prior to participation, written informed consent was obtained from every individual involved.

## Funding

The study did not receive funding.

## Declaration of competing interest

The authors report no declarations of interest.
